# Automated Collection of Real-Time Alerts of Citizens as a Useful Tool to Continuously Monitor Malodorous Emissions

**DOI:** 10.3390/ijerph13030263

**Published:** 2016-02-26

**Authors:** Magda Brattoli, Antonio Mazzone, Roberto Giua, Giorgio Assennato, Gianluigi de Gennaro

**Affiliations:** 1 Apulia Regional Agency for Environmental Prevention and Protection, Corso Trieste 27, 70126 Bari, Italy; m.brattoli@arpa.puglia.it (M.B.); a2.mazzone@arpa.puglia.it (A.M.); r.giua@arpa.puglia.it (R.G.); dg@arpa.puglia.it (G.A.); 2 Department of Biology, University of Bari Aldo Moro, Via Orabona 4, 70126 Bari, Italy

**Keywords:** noses of citizens, dynamic olfactometry, odor annoyance, real-time sampling, remote monitoring system

## Abstract

The evaluation of odor emissions and dispersion is a very arduous topic to face; the real-time monitoring of odor emissions, the identification of chemical components and, with proper certainty, the source of annoyance represent a challenge for stakeholders such as local authorities. The complaints of people, often not systematic and variously distributed, in general do not allow us to quantify the perceived annoyance. Experimental research has been performed to detect and evaluate olfactory annoyance, based on field testing of an innovative monitoring methodology grounded in automatic recording of citizen alerts. It has been applied in Taranto, in the south of Italy where a relevant industrial area is located, by using Odortel^®^ for automated collection of citizen alerts. To evaluate its reliability, the collection system has been integrated with automated samplers, able to sample odorous air in real time, according to the citizen alerts of annoyance and, moreover, with meteorological data (especially the wind direction) and trends in odor marker compounds, recorded by air quality monitoring stations. The results have allowed us, for the first time, to manage annoyance complaints, test their reliability, and obtain information about the distribution and entity of the odor phenomena, such that we were able to identify, with supporting evidence, the source as an oil refinery plant.

## 1. Introduction

Olfactory annoyance is felt to be an indicator of an unhealthy environment by the population [1]; as this considerably complicates the overall assessment of odor pollution, more innovative methodologies compared to simple air quality monitoring are needed [2]. The continuous exposure to odor emissions could cause undesired reactions such as physiological symptoms (respiratory problems, nausea, headache, eye irritation) and psychological stress (anxiety, depression) [3,4,5,6]. Moreover, olfactory pollution could also produce effects about social context, such as impairment of the environment quality, damages to properties, harm or discomfort, injury, loss of enjoyment of normal use of property, or interference with business activities [1]. Therefore, the acceptability of industrial plants and agricultural farms, very often a source of bad odors, is limited and their closer and closer proximity to residential areas leads to several citizen’s complaints [7,8]. Due to the strict connection of odor pollution to human perception, the only application of standardized methodologies for the monitoring and control of emissions is not sufficient to provide useful information about the overall understanding of odor phenomena [9]. For this reason, where industrial plants causes olfactory annoyance to the population, it should be fundamental to endorse the role of people participation to assess exposure to odors [10,11]. In recent years, even international regulations about odor emissions tend to consider the employment of human assessors as a valuable method to assess the odor impact of an industrial plant on the territory. For example, German VDI 3940 standardizes a method of field inspection by using trained assessors, applied by several researchers in order to get quantitative indexes of annoyance [12,13,14,15,16]. However, this approach is time-consuming and requires some months of investigation before obtaining valuable results. Another common qualitative investigation to evaluate the perception of odors within an area of study can be conducted by means of administration of questionnaires addressed to the residents [17,18,19,20,21,22,23,24], the purpose of which is to understand the level of stress within a community and to characterize the occurrence of the events. Even in this case, the evaluation requires long periods of investigation to achieve statistically significant results, necessary to overcome the intrinsic subjectivity of resident responses. Generally, people exposed to annoyance variously address their worries to the local authorities (municipalities, police, environmental agencies, *etc.*), who are often overwhelmed by frequent complaints; they should evaluate the reliability and objectivity of the complaints and identify the potential source, in order to start monitoring activity and give prompt answers. To achieve this task, different critical aspects have to be managed: the collection of complaints in a systematic way, the verification of odor events in the field, and their measurement. Above all, the last two aspects represent limiting factors, since a variable period of time always passes from the signal to the operative intervention of environmental agencies and it might be that the odor is no longer detectable. That makes it difficult to prove the reliability of complaints and identify the cause of the event [25].

This paper focuses on the first application of an automatic system, called Odortel^®^, developed to collect citizen alerts and record them in real time. The Apulia Regional Agency for Environmental Prevention and Protection—ARPA has been testing the performance of this system in a relevant and complex industrial site. The recording of olfactory perception of human receptors, accurately organized and georeferenced, has allowed us to view the spatial distribution of the odorous phenomenon immediately and, over time, to quantify its true extent. Moreover, this information has been correlated with meteorological data, especially wind direction, in order to evaluate the reliability of alerts and screen the potential source. The study has also integrated the automatic recording of citizen annoyance alerts with automated samplers, located in different parts of the territory, aiming to sample, in real time, odorous air to be analyzed by dynamic olfactometry [26]. The information provided by citizens, about time and duration of events has been also compared with trends in odor marker compounds recorded by air quality monitoring stations.

## 2. Experimental Section

### 2.1. Site Description

**Figure 1:**
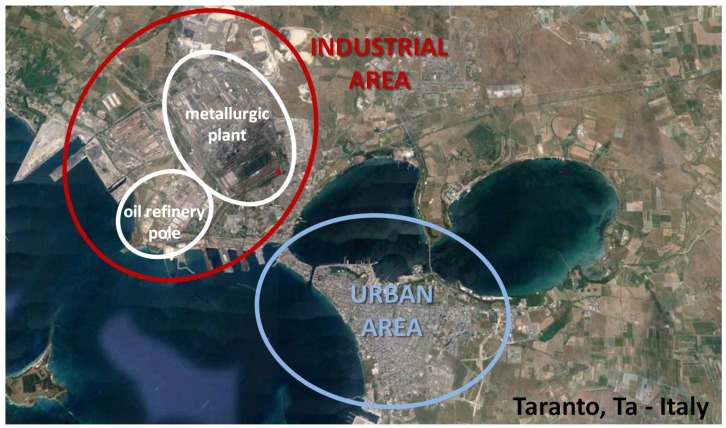
Summary map of Taranto city, with indication of industrial and urban areas of Taranto.

The experimental research, performed to detect and evaluate olfactory annoyance by a new innovative monitoring methodology based on recorded citizen alerts, has been applied in the area of Taranto, a city in the south of Italy. The urban area of Taranto is seriously afflicted by environmental pollution arising from an industrial area close to the city. This industrial area hosts, among others, a well-known district of steel production, the biggest metallurgic center in Europe and already considered a significant source of pollution for the city. In this context (already critical because of significant atmospheric emissions), the industrial area of Taranto hosts an important oil refinery plant that, potentially, can also produce olfactory annoyance, due to its typical activities and emitted compounds, such as H_2_S and mercaptans (Figure 1). In particular, potential odor sources can be identified, according to the productive cycle of the refinery: plant units for the desulfurization process, n. 130 tanks for the storage of hydrocarbon products, a wastewater treatment plant, and loading and unloading areas.

The olfactory annoyance arising from it represents a further worsening of quality of life for the population exposed, which severely limits the social acceptability of the industrial plants.

### 2.2. Integrated System Description

The applied methodology is based on the direct involvement of a sample of the resident population; it consists in an automatic remote system, which records the olfactory perception of human receptors and collects representative samples in real time. The main features of the approach used are: (a) systematization and digitalization of telephone complaints; (b) real-time map displaying; and (c) real-time remote sampling. The system comprises a communication platform of the olfactory discomfort, telephone based, through which each trial participant, appropriately coded, indicates the odor event perception and its intensity (via telephone keypad) on a scale of three levels, displayed with different color codes on the web platform: 1—faint odor (color code green); 2—distinct odor (color code yellow); 3—very strong odor (color code red). A database collects the call recordings and allows the real-time displaying of the warnings. The graphic interface system allows us to query the database and get information about date, time, and number of reports both in a synoptic frame and on the map. The overrun of appropriate thresholds, based on the number of calls for index of intensity in a defined range of time, produce the real-time activation of a sampling device, located on the experimental area, that collects air samples to analyze, according to the technical standard EN 13725: 2003 [26]. The sampling system is based on the so called “lung principle,” according to which a sampling bag is placed in a rigid and opaque container, and the air removed from it using a vacuum pump. The unit is able to activate the remote sampling system by means of a GSM telephone dialer that handles text messages (SMS). After that, the signal is received and, when the sampling is finished, the unit promptly alerts the technicians, via SMS, to collect the sample. The sampling system is equipped with two independent lines, which can be activated either simultaneously or in sequence. The two sampling lines are kept in a cab, equipped with an automatic temperature control system (heating and cooling) in order to avoid any condensation phenomena.

### 2.3. Steps of the Experimental Activities

#### 2.3.1. Recruitment of the Sample Population

**Figure 2:**
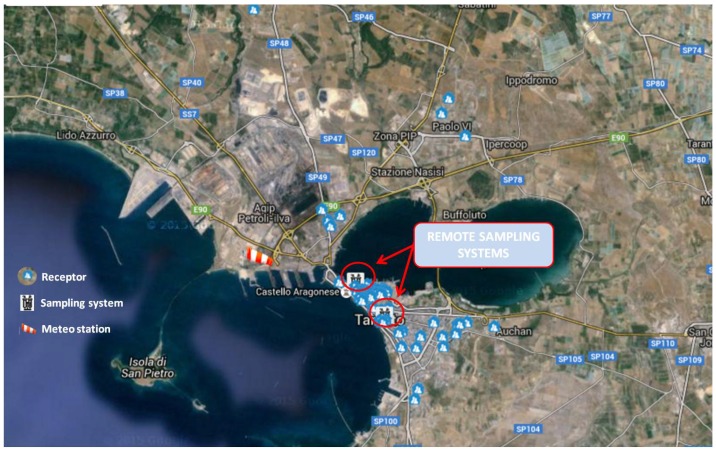
Location of receptors, remote sampling systems, and meteorological station on map of Taranto.

The experimental work was started in November 2013 and currently involves 65 receptors, who voluntarily joined in the project; this number has been increasing over time. The position of each receptor has been georeferenced and an identification code has been assigned to each of them in order to allow the call recording and the participation in the project. Figure 2 shows the localization of the receptors and of the remote sampling systems (described in Section 2.3.2) on the map of Taranto. It can be noticed that most of the receptors are located in the downtown area of the city.

#### 2.3.2. Remote Monitoring System

Two remote automatic sampling systems with two independent sampling lines have been placed in Taranto. One is placed in Piazza Garibaldi, close to the air quality control units of ARPA Puglia, and the other at the Hospital SS Annunziata. The two homologous lines of the two samplers, line 1 and line 2, are activated simultaneously as a result of exceeding the trigger threshold set. The samples are collected in polyethylene terephthalate bags, commercially known as Nalophan^®^.

Figure 2 shows the location of receptors, remote sampling systems, and automatic weather station. In particular, the weather station is sited at the local Department of ARPA Puglia (ex Testa Hospital—Contrada Rondinella), near the refinery oil plant in the industrial area of Taranto. It is equipped with seven sensors for the measurement of specific parameters: temperature, relative humidity, precipitation, wind speed, wind direction, atmospheric pressure, and global solar radiation.

#### 2.3.3. Sampling Protocol

The activation of the remote sampling takes place when properly chosen thresholds are exceeded; these thresholds are set taking into account the number of receptor warnings (distinguished according to the intensity index) recorded in one hour. Moreover, during the experimental work, the activation thresholds have been re-modulated, in order to make them more severe and so more representative of odorous events. The indication of successful sampler activation and of sample bags filling is sent automatically, via SMS, to technicians involved in the withdrawing of the sample for the analysis. The sampling protocol also provides for the withdrawal of environmental samples, called “blank”, collected when technicians pick up the odorous samples, which occurs some hours later than the activation of the automatic remote sampling, in order to verify the levels of odor environmental background in a different moment from the occurrence of an odor event.

### 2.4. Odor Analysis by Dynamic Olfactometry

The samples have been analyzed by dynamic olfactometry, which is the only standardized method for objective and quantitative determination of the odor concentration in a gas sample, according to the technical standard EN 13725: 2003. Odor samples have been tested at the olfactometric laboratory of University of Bari, a partner in the experimental project, equipped with an adequate olfactometric room, in compliance with the requirements of EN 13725: 2003. The laboratory employs an olfactometer ECOMA GmbH Mannebeck TO8 model, with four workstations for contemporary tests and mastered by a PC equipped with control software only. As required by European regulations (EN 13725), the olfactometric laboratory complies with the quality requirements in terms of accuracy and repeatability.

## 3. Results and Discussion

The systematizing of citizen complaints by means of the Odortel^®^ system has provided fundamental information to evaluate the entity of olfactory annoyance and to spatially and temporally characterize the events in order to identify the source. In this section, the results of the experimental activity (from the beginning of the project to October 2015) are shown for the specific application in Taranto; the research is currently in progress.

### 3.1. Distribution of Citizen Complaints

An important first indication about the extent of olfactory annoyance perceived by the population can be learned by analyzing the number of calls recorded and their distribution over the period of activity. Overall, 1140 phone calls have been recorded by Odortel^®^ system, divided into three intensity levels, as shown in Table 1.

As reported in Table 1, the majority of calls belong to level 3, which denotes a strong perception of odor phenomenon in the community. Furthermore, the number of alerts in 2015 has been much lower than in 2014, a tendency confirmed by the same receptors who, during the periodic meetings planned to monitor the advancement of the project, declared a decrease in odor events in the first part of the year.

#### Spatial Distribution

**Figure 3:**
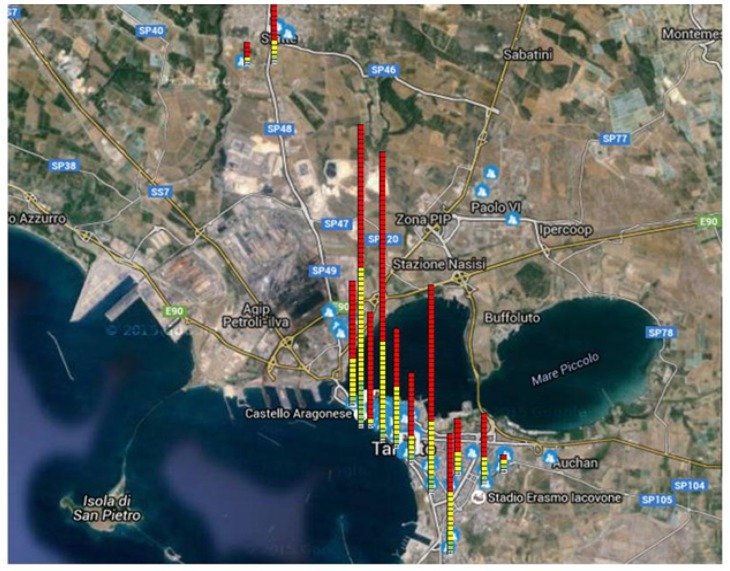
Graphic representation of the most representative warnings on a map. Green (level 1), yellow (level 2), and red (level 3) squares indicate the intensity level of complaints of most active receptors.

The automatic registration of phone complaints and the possibility of immediately viewing their position on a map constitute two more advantages of the methodological approach. In fact, in order to totally cover the surroundings of the industrial sites, the receptors are located in different areas of the territory; the number and the frequency of their complaints allow us to study the spatial distribution of odor perception and identify the most critical areas. The collected data have shown that the most copious complaints derive from residents living in the center of the city, close to the coastal area, as shown in Figure 3.

### 3.2. Evaluation of the Reliability of Citizen Complaints

The reliability and truthfulness of citizen complaints are key elements to guarantee the good functionality of the system and to perform a first validation of the results. As previously described, the project is based on volunteers, most of them dissatisfied with air quality, so the methodology has to be planned in order to avoid any risks of obtaining flawed results and to prove the reliability of citizen indications. To this end, wind direction, recorded during the alerts, is a good indicator to establish downwind and upwind meteorological conditions for receptors, compared to the position of the industrial area, where the source of odor emissions is sited. For each complaint registered by the Odortel system, the wind direction and speed data have been collected and combined with the odor intensity level communicated by each receptor. Bivariate polar plots have been created to relate the odor intensity perceived by receptors located in the center of Taranto (who reported the most complaints) with the direction and speed of the wind.

**Figure 4:**
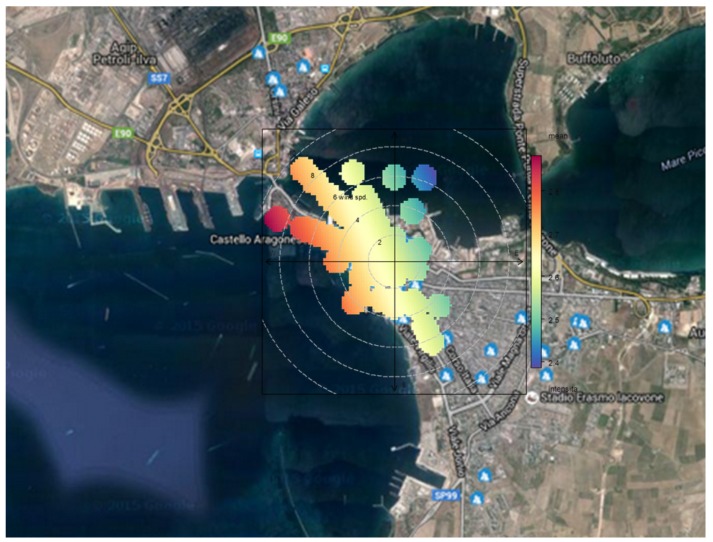
Polar plot of the relationship between the perceived odor intensity (from red—very strong odor perception to blue—low odor perception) and the wind parameters (direction and speed).

Figure 4 displays the results of this processing in graphic form; the colored surfaces are radially disposed according to prevalent wind direction and the chromatic scale indicates the relative value of odor intensity. The graph shows that the higher intensity level, very strong odor, occurred when receptors were in downwind conditions (North–West quadrant) with respect to the oil refinery pole. This evidence constitutes a first validation of the system and confirms the reliability of receptors’ indications.

### 3.3. Olfactometric Results

**Figure 5:**
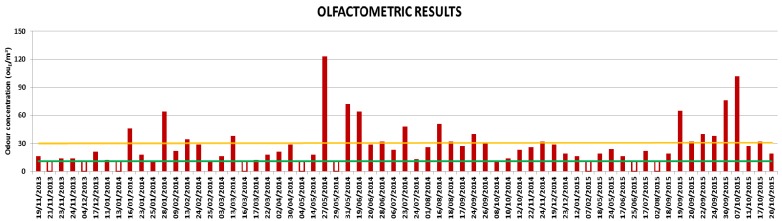
Olfactometric results, expressed in ou_E_/m^3^, measured during odor events. The bars indicate the maximum odor concentration measured during each odor event. The graph does not report the events for which the results were not considered valid and displays only the maximum odor concentration in cases where two activations happened at different time intervals of the same day.

The exceedance of the chosen routine (number of calls per index of intensity in one hour) automatically activates the two remote samplers, located in the area, in order to collect samples of odorous air, then analyzed according to dynamic olfactometry methodology. Overall, 70 odor events were detected by remote sampling up to October 2015, 50 of which were in 2014 and the remainder in 2015. So, a significant decrease has been verified in 2015, as confirmed even by receptor feelings, but it has to be underlined that half of the odor events in 2015 occurred in September–October 2015, indicating a renewed exacerbation of the phenomenon. Figure 5 displays the maximum odor concentrations, expressed in ou_E_/m^3^, measured for each odor event. The empty bars indicate the events for which no odor concentration has been objectively detected; 11 ou_E_/m^3^ (shown with a green line) represents the instrumental quantification limit for the olfactometric laboratory. The orange line in the graph corresponds to 30 ou_E_/m^3^; this value has been chosen to define a limit to identify the most significant odor events. The choice of this criterion is derived from the analysis of the olfactometric concentrations measured for “blank” samples, as described below.

In Table 2, the odor concentration distribution of “blanks” is reported. Thirty-four percent of the blank concentrations are lower than the instrumental quantification limit and 76% lower than 20 ou_E_/m^3^, underlining that the background odor is not so relevant. So, the criterion of 30 ou_E_/m^3^ to define the significance of an odor event was decided upon by considering a value approximately equal to three times the minimum quantification limit and it has been studied for this specific application.

**Figure 6:**
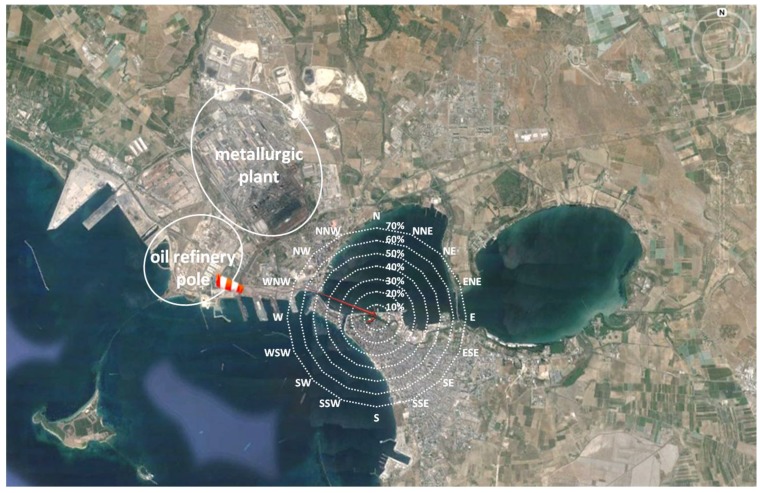
Frequency of occurrence related to wind directions recorded during the most significant odor events. In the map the location of a weather station is identified with this icon: 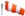.

The analysis of wind directions, recorded during the sampling of all odor events, causing the remote samplings, revealed that the wind blew from WNW to NW in 60% of all cases with a prevalence of 50% for WNW, clearly ascribable to the oil refinery pole, while it blew from NNW, indicating the metallurgic plant, only in 10% of events. In particular, when only the most significant events were considered, the frequency related to the WNW–NW range increases up to 70% (with a prevalence of about 65% for WNW) while that related to NNW decreases to 5%, as shown in Figure 6. This information has been valuable to attribute odor annoyance to the oil refinery pole.

Moreover, during the analysis, the examiners qualitatively described the odor perceived; for significant events, they defined the odor as similar to “rotten eggs” or “gas”, typical descriptors of odors deriving from oil refineries. The number of significant odor events, out of 70 events in total, produced by remote sampling, suggests that the odor concentrations measured could be underestimated with regard to population perception. In fact, it is important to underline that, taking into account the results of the recent scientific studies, several causes could contribute to the general degradation of sample in bags [27,28]. The reduced lifetime of sulfur compounds [29,30] probably emitted by the investigated source means we should consider the measured odor concentrations as precautionary levels compared to that actually perceived. Moreover, Nalophan bags could be very permeable to some strongly odorous compounds, characterized by small size and/or particular physico-chemical properties, such as ammonia and hydrogen sulfide [31,32].

### 3.4. Integration of Air Quality Data with Odortel^®^ System

**Figure 7:**
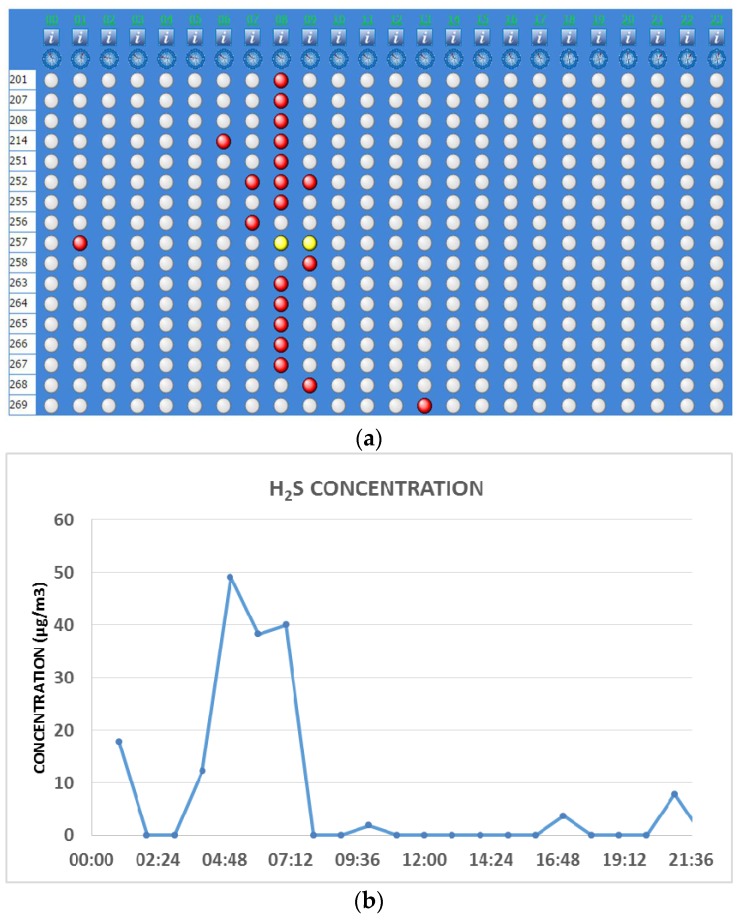
(**a**) Synoptic hourly visualization of complaints recorded by Odortel^®^ on 1 August 2014 (each row refers to the annoyed receptor and each column indicates each hour of the day); (**b**) Analytical responses of H_2_S concentrations, recorded on 1 August 2014.

To support the evidence shown by the application of Odortel^®^, possible correlations with air quality data (in particular H_2_S) recorded by monitoring stations located in different parts of Taranto have been investigated. H_2_S concentration peaks corresponding to odorous events perceived by the population have been verified. As an example, in Figure 7a,b an odor event that occurred on 1 August 2014 is illustrated; the correlation between odor perception signaled by the population and H_2_S concentration data at the monitoring station close to the oil refinery clearly revealed a causal relationship existing between the supposed source and residents’ perception. Figure 7a shows the synoptic hourly visualization of complaints recorded by Odortel^®^ and the trends in analytical responses of H_2_S concentrations (Figure 7b), which show increases in the same time range. The first three complaints were recorded at 6:41 a.m., 7:00 a.m., and 7:55 a.m. (local time); the other complaints occurred in the range 8:00–9:00 (local time). The H_2_S concentrations were measured at the monitoring station located close to the refinery. The delay between the H_2_S peak and the complaints is compatible with the distance from the refinery to the center of the city. Moreover, the odor event occurred in the first part of the morning, when most of the population was still asleep and probably was not yet able to perceive the odor.

## 4. Conclusions

A successful management of complex cases of olfactory annoyance cannot disregard the key role of social participation, to be considered as a tool to characterize the phenomenon and correctly identify the emitting source. To that end, it is necessary to start from the perception of the annoyed population, overcoming the subjectivity of human feelings. To achieve this task, the numerous complaints declared by the population have to be collected in an organized way, saving the information about time and the position of alerts. An automatic system, such as Odortel^®^, allows us to gain useful information related to the incidence and distribution of odor phenomena. Moreover, the digitizing of receptors’ complaints leads us to perform further elaborations aimed at validating the reliability of the alerts and evaluating the potential odor source on the ground. The analysis of wind direction represents a first step of validation, as we are able to identify areas under downwind conditions. Citizen real-time alerts can be used to activate remote sampling at the same moment as the complaints, as described in this research project, but also to correlate with continuous air quality monitoring data, referring to some chemical markers of the emissions. The first application of the system has demonstrated its effectiveness and real applicability at a complex site, the Taranto industrial area; in fact, it has been possible to identify a causal relationship between the citizen annoyance and the oil refinery emissions with scientific support and evidence, essential factors for the authorities to apply controlling measures and actions towards the emitting source. Furthermore, the active involvement of the exposed population also promotes awareness about odor phenomena.

## Figures and Tables

**Table 1:** Number of complaints/intensity level recorded in the period November 2013–October 2015.

Number of Complaints/Intensity Level			
	November 2013–December 2014	2015 (through October)	Total
FAINT ODOR (LEVEL 1)	68	54	122
DISTINCT ODOR (LEVEL 2)	251	120	371
VERY STRONG ODOR (LEVEL 3)	460	187	647
TOTAL	779	361	1140

**Table 2:** Distribution of odor concentration for “blank” samples.

Odor Concentration (ou_E_/m^3^)	Frequency (Number of Events)	Percentage (%)
<11	32	34
11–20	40	42
21–30	10	11
31–40	5	5
41–50	4	4
51–60	0	0
61–70	2	2
71–80	0	0
81–90	2	2
